# Species-specific relationships between deep sea sponges and their symbiotic *Nitrosopumilaceae*

**DOI:** 10.1038/s41396-023-01439-4

**Published:** 2023-05-31

**Authors:** Alessandro N. Garritano, Marwan E. Majzoub, Bárbara Ribeiro, Taissa Damasceno, Fluvio Modolon, Camila Messias, Caren Vilela, Gustavo Duarte, Lilian Hill, Raquel Peixoto, Torsten Thomas

**Affiliations:** 1 Centre for Marine Science and Innovation, School of Biological, Earth and Environmental Sciences, Faculty of Science, The University of New South Wales, Kensington, NSW 2052 Australia; 2 King Abdullah University of Science and Technology, Biological and Environmental Science and Engineering Division, Thuwal, Saudi Arabia; 3 Universidade Federal do Rio de Janeiro, Instituto de Biologia, Departamento de Microbiologia Paulo de Goes, LEMM Laboratory, Rio de Janeiro, Brazil

**Keywords:** Microbial ecology, Symbiosis

## Abstract

Sponges thrive in the deep, dark and nutrient-depleted ocean and may rely on microbial symbionts for carbon acquisition and energy generation. However, these symbiotic relationships remain largely unexplored. In this study, we analyze the microbiome of deep-sea sponges and show that ammonia-oxidizing archaea (AOA) of the family *Nitrosopumilaceae* make up at least 75% of the microbial communities of the sponges *Aphrocallistes sp*., *Farrea* sp. and *Paratimea* sp.. Given the known autotrophic metabolism of AOAs, this implies that these sponge holobionts can have the capacity for primary production in the deep-sea. We also show that specific AOA lineages are highly specific towards their hosts, hinting towards an unprecedent vertical transmission of these symbionts in deep-sea sponges. Our results show that the ecology and evolution of symbiotic relationships in deep-sea sponge is distinct from that of their shallow-water counterparts.

## Introduction

Symbiotic relationships with microorganisms are fundamental for sponges thriving in oligotrophic environments, such as the deep sea [1, 2]. In this environment, sponges could rely on chemolithoautotrophic microorganisms to obtain organic carbon [3]. Carbon fixation in such environments can be driven by the energy-efficient 3-hydroxypropionate/4-hydroxybutyrate (HP/HB) cycle, which has been found so far in all members of the ammonia-oxidizing archaea (AOA) belonging to the family *Nitrosopumilaceae* (phylum *Thermoproteota*, class *Nitrososphaeria*, order *Nitrososphaerales*) [4].

Substantial efforts have been recently made to analyze the microbial communities of deep-sea sponges [5, 6], although the importance of *Nitrosopumilaceae* has been overlooked. Here, we compare the relative abundances of amplicon sequencing variants (ASVs) assigned to this family across 107 different seawater, sediment and sponge samples collected from deep waters (~700 m) at the Campos Basin, Southeastern Brazil (22°38’S/40°25’W) (Supplementary Table S1). ROV-based sampling, individually contained transport to the surface and careful sample processing allowed us to generate sample-specific microbial community profiles (see Materials and Methods in Supplementary Text) to better define the fundamental associations between *Nitrosopumilaceae*, their hosts and the environment. Our results suggest that *Nitrosopumilaceae* are dominant in some deep-sea sponges and form highly species-specific relationship with their host

## Results and discussion

**Fig. 1:**
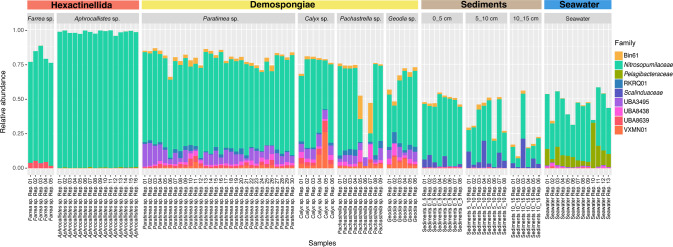
Taxonomic profiles of bacterial and archaeal communities in deep-sea sponges, sediment and seawater samples. The profiles are presented as relative abundances (%), only showing taxa with am average >1%. Sediment samples were presented according to the different depths. Rep. stands for replicate.

ASV-based analysis for the V4 region of the 16S rRNA gene [7] of 72 samples from the sponge classes Hexactinellida (*Farrea* sp. and *Aphrocallistes sp*.) and Demospongiae (*Paratimea* sp., *Calyx* sp., *Geodia* sp., *Pachastrella* sp.) (Supplementary Figs. S1–6) showed distinct community profiles when considering sponge class or species (Supplementary Fig. 7) and a high relative abundance of sequences assigned to the family *Nitrosopumilaceae* (Fig. 1). Among the Hexactinellida, *Nitrosopumilaceae* ASVs represent, on average, 76.98 ± 0.04% of all reads in *Farrea* sp. and 97.70 ± 0.01% in *Aphrocallistes* sp., which was significantly higher than in sediments (average relative abundance 30.39 ± 14.18%, PERMANOVA *F*_*2*_ = 67.37, *R*^2^ = 0.63, *p* = 0.006) and seawater (average relative abundance 33.90 ± 11.56%, PERMANOVA *F*_*2*_ = 50.23, *R*^2^ = 0.61, *p* = 0.006). For Demospongiae, *Nitrosopumilaceae* ASVs constitute an average of 63.74 ± 5.10% of all reads in *Paratimea* sp., 53.15 ± 10.94% in *Calyx* sp., 42.54 ± 26.78% in *Pachastrella* sp. and 43.84 ± 13.97% in *Geodia* sp. The relative abundance of *Nitrosopumilaceae* ASVs within *Paratimea* sp. is significantly higher than in sediments (PERMANOVA *F*_*2*_ = 55.15, *R*^2^ = 0.52, *p* = 0.028).

**Fig. 2:**
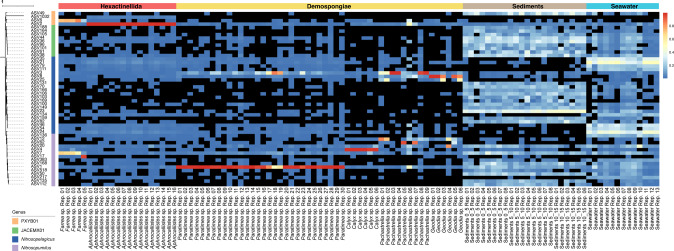
ASV-level analysis for the distribution of *Nitrosopumilaceae* sequences in deep-sea sponges, sediments and seawater samples. The relative square-rooted abundances (heat-scale) of the 50 most abundant ASVs assigned to the *Nitrosopumilaceae* family are shown. Black squares in the heatmap refers to the ASV not being detected in the sample. The tree of the left is rooted with *Nitrososphaera viennensis* (not shown). Scale bar represents the tree scale. Rep. stands for replicate.

More detailed analysis showed that the genus *Nitrosopumilus* dominates the samples from Demospongiae (up to 99.25% of all *Nitrosopumilaceae* reads), while the placeholder genus PXYB01 is more abundant in the Hexactinellida samples (up to 99.21% of all *Nitrosopumilaceae* reads) (Fig. 2, Supplementary Table 1).

Our data also show that a single ASV (ASV2 – placeholder genus PBXY01) represents on average 98.17 ± 0.08% of the *Nitrosopumilaceae* reads across all 16 *Aphrocallistes* sp. samples (Fig. 2). To the best our knowledge, this is the first time that a single Nitrosopumilaceae ASV has been found to be present in such a high proportion in an environmental sample. A similar dominance is also observed for ASV1 (genus *Nitrosopumilus*) in *Paratimea* sp., which account for an average relative abundance of 91.31 ± 17.50%. Similarily, ASV6 constitutes, on average, 47.74 ± 2.43% of all *Nitrosopumilaceae* reads of *Farrea* sp. samples. This dominance of one ASV in the sponge samples contrasts the *Nitrosopumilaceae* community in seawater or sediments, where at least 7 and 5 ASVs, respectively, are needed to make up 50% of *Nitrosopumilaceae* reads. Finally, the ASVs found in high proportions in sponges are generally undetected or only found in low relative abundances in seawater and sediment samples supporting the notion of a high degree of specificity.

Functional redundancy of symbiotic microorganisms involved in nitrogen and carbon cycles has been noted for sponge holobionts [8]. However members of the family *Nitrosopumilaceae* seem to have an exclusive role in ammonium oxidation in sponges [9]. By oxidizing ammonium or urea, these microorganisms may contribute to detoxification processes in the sponge holobiont [10]. Furthermore, ammonium oxidation can generate the reductive power for carbon fixation and fixed organic carbon could ultimately be transferred to the sponge host, similar to what has been shown for cyanobacterial photosynthates in shallow-water sponges [11].

In contrast to recent studies [5], our results show that *Nitrosopumilaceae* symbionts have a species-specific relationship with their host, hinting to their possible vertical transmission. Vertical transmission is unlikely to be strong driver of community composition in variable environments and indeed is not very common in shallow-waters or near-shore sponges [12]. In contrast, the environmental stability of the deep sea [13] would allow, as shown here, for highly specific and possibly vertically transmitted symbioses to evolve. Our results also suggest the importance of chemolithoautotrophy through members of the *Nitrosopumilaceae* family in deep-sea sponges and further work will reveal how this contributes to the primary productivity of deep-sea communities.

## Supplementary information

Supplementary Materials and Methods

Supplementary Figures

Supplementary Table 1

Supplementary Table 2

## Data Availability

The raw reads have been deposited in the NCBI BioProject database (https://www.ncbi.nlm.nih.gov/bioproject/) under BioProject accession number PRJNA930637. All other study data are included in the article and/or supporting information files.
