# Supplementary material for: Species-specific relationships between deep sea sponges and their symbiotic Nitrosopumilaceae
Source: ISME J. 2023 May 31;17(9):1517–9. doi: 10.1038/s41396-023-01439-4 (PMC10432484; doi:10.1038/s41396-023-01439-4)
Supplement: Supplementary file 1 — Supplementary Materials and Methods [file 41396_2023_1439_MOESM1_ESM.docx]

**Materials and methods:**

Seawater, sediment and sponge sampling

Samples were collected during a ship-based expedition at the Campos Basin, Rio de Janeiro, Brazil, during late August/early September 2021. Five sponge grounds in this basin were visited (Supplementary Table 2) and 72 sponge samples (50 Demospongiae and 26 Hexactinellida) were collected by a remotely operated vehicle. Sponge samples were transferred into individual, customed-built boxes, which were sealed but allowed for pressure equilibration during ascend. Upon surfacing, subsamples of each sponge were cut with sterile equipment and washed three times with sterile seawater to remove loosely-associated microorganisms. Samples were then immediately frozen in liquid nitrogen. Fifteen seawater samples were collected with GO-FLO bottles (General Oceanics, Inc., Miami, FL, USA) approximately five meters above the sampling sites and immediately filtered onto 0.22 µm filters after surfacing. The filters were placed into sterile cryovials and snap frozen in liquid nitrogen. Twenty-two sediment samples were collected with box-cores in the vicinity of the sampling sites. After surfacing, the sediment samples were stratified into three different depths (0-5 cm, 5-10 cm, and 10-15 cm), stored in sterile Whirl-Pak® bags and snap frozen in liquid nitrogen.

*DNA extraction, 16S rRNA gene sequencing and processing*

DNA was extracted using the DNeasy PowerSoil Pro Kit (Qiagen, Hilden, Germany) according to the manufacturer’s instruction. The V4 hypervariable region of the 16S rRNA gene was amplified using the modified primer pairs 515F (Parada) and 806R (Apprill) [1, 2] and sequenced on a MiSeq platform (Illumina, San Diego, CA, USA) at the Ramaciotti Centre for Genomics (University of New South Wales, Sydney, Australia). Raw reads were deposited at the National Centre for Biotechnology Information (NCBI) under BioProject ID PRJNA930637.

Raw sequences were quality-filtered using Trimmomatic (version 0.38) in a sliding window of 4 bp and reads with a quality score below 15 or shorter than 100 bp were discarded [3, 4]. Paired-end reads were merged using USEARCH (version 11.0.667) [5] with a minimum 8 bp overlap and reads shorter than 260 bp or longer than 300 bp were removed. The UNOISE3 algorithm [5] was used to cluster, denoise and generate the amplicon sequence variants (ASVs). Chimeras were removed using the UCHIME3 and the SILVA version 38 database (high confidence mode) [5]. Taxonomy was assigned to the ASVs sequences using the BLCA algorithm [6] (identity and coverage intervals set to 95%-100%) against a filtered version of the Genome Taxonomy Database (GTDB version 207), in which all sequences shorter than 1000bp were removed [7]. One water sample and one sponge sample were removed from the dataset due to a low number of reads (<5,000). Data were normalized for read counts using the DESeq2 package [8]. All non-bacterial and non-archaeal ASVs sequences were removed, and reads were mapped to the remaining ASVs sequences to estimate the relative abundances.

The data was visualised using R (version 4.0.2). The sequences for the most abundant ASVs assigned to the *Nitrosopumilaceae* family were retrieved and aligned using MUSCLE (version 3.8) [9]. A phylogenetic tree was built using IQ-tree (version 1.6.12)[10] with 1,000 bootstraps.

*Sponges molecular identification*

Sponges were taxonomically classified by Sanger sequencing part of the 28S rRNA gene with the primer pairs C2 and D2 [11], or part of the COI gene using either primer pairs dgLCO1490 and dgLCO2198 [12] or CO1porF1 and CO1porR1 [13]. Sequences were manually checked for quality. Voucher sequences for relevant sponge genera were retrieved from NCBI and aligned using MUSCLE (version 3.8) [9]. Sponges were taxonomically classified based on phylogenetic trees that were built including suitable reference sequences using IQ-tree (version 1.6.12) [10] with 1,000 bootstraps (Supplementary Figures 1-6). Taxonomy was derived by monophyly of sample sequences with reference sequences.

**References for Materials and Methods:**

1. Parada AE, Needham DM, Fuhrman JA. Every base matters: Assessing small subunit rRNA primers for marine microbiomes with mock communities, time series and global field samples. *Environ Microbiol* 2016; **18**:1403–1414.

2. Apprill A, Mcnally S, Parsons R, Weber L. Minor revision to V4 region SSU rRNA 806R gene primer greatly increases detection of SAR11 bacterioplankton. *Aquat Microb Ecol* 2015; **75**:129–137.

3. Bolger AM, Lohse M, Usadel B. Trimmomatic: A flexible trimmer for Illumina sequence data. *Bioinformatics* 2014; **30**:2114–2120.

4. Wemheuer B, Wemheuer F. Assessing Bacterial and Fungal Diversity in the Plant Endosphere 2017;**1539**:75–84.

5. Edgar RC. UNOISE2: improved error-correction for Illumina 16S and ITS amplicon sequencing. *bioRxiv* 2016;081257.

6. Gao X, Lin H, Revanna K, Dong Q. A Bayesian taxonomic classification method for 16S rRNA gene sequences with improved species-level accuracy. *BMC Bioinformatics* 2017;**18**:1–10.

7. Parks DH, Chuvochina M, Chaumeil PA, Rinke C, Mussig AJ, Hugenholtz P. A complete domain-to-species taxonomy for Bacteria and Archaea. *Nat Biotechnol* 2020;**38**:1079–1086.

8. Love MI, Huber W, Anders S. Moderated estimation of fold change and dispersion for RNA-seq data with DESeq2. *Genome Biol* 2014;**15**:550.

9. Edgar RC. MUSCLE: Multiple sequence alignment with high accuracy and high throughput. *Nucleic Acids Res* 2004;**32**:1792–1797.

10. Nguyen LT, Schmidt HA, Von Haeseler A, Minh BQ. IQ-TREE: A fast and effective stochastic algorithm for estimating maximum-likelihood phylogenies. *Mol Biol Evol* 2015;**32**:268–274.

11. Chombard C, Boury-esnault N, Tillier S. Reassessment of Homology of Morphological Characters in Tetractinellid Sponges Based on Molecular Data. *Syst Biol*. 1998;**47**:351–366.

12. Meyer CP, Geller JB, Paulay G. Fine scale endemism on coral reefs: Archipelagic differentiation in turbinid gastropods. *Evolution (N Y)* 2005;**59**:113–125.

13. Erpenbeck D, Hooper JNA, Wörheide G. CO1 phylogenies in diploblasts and the ‘Barcoding of Life’ - Are we sequencing a suboptimal partition? *Mol Ecol Notes* 2006;**6**:550–553.
