# Supplementary material for: Species-specific relationships between deep sea sponges and their symbiotic Nitrosopumilaceae
Source: ISME J. 2023 May 31;17(9):1517–9. doi: 10.1038/s41396-023-01439-4 (PMC10432484; doi:10.1038/s41396-023-01439-4)

Supplementary Figure 1: Phylogenetic tree for *Aphrocallistes* sp. samples based on part of the COI gene using primer pairs dgLCO1490 and dgLCO2198. Reference sequences were obtained from NCBI. The tree is rooted with *Paramuricea clavata* (not shown here). Organisms in red represent voucher sequences deposited in NCBI. Scale bar represents the tree scale. Rep. stands for replicate.


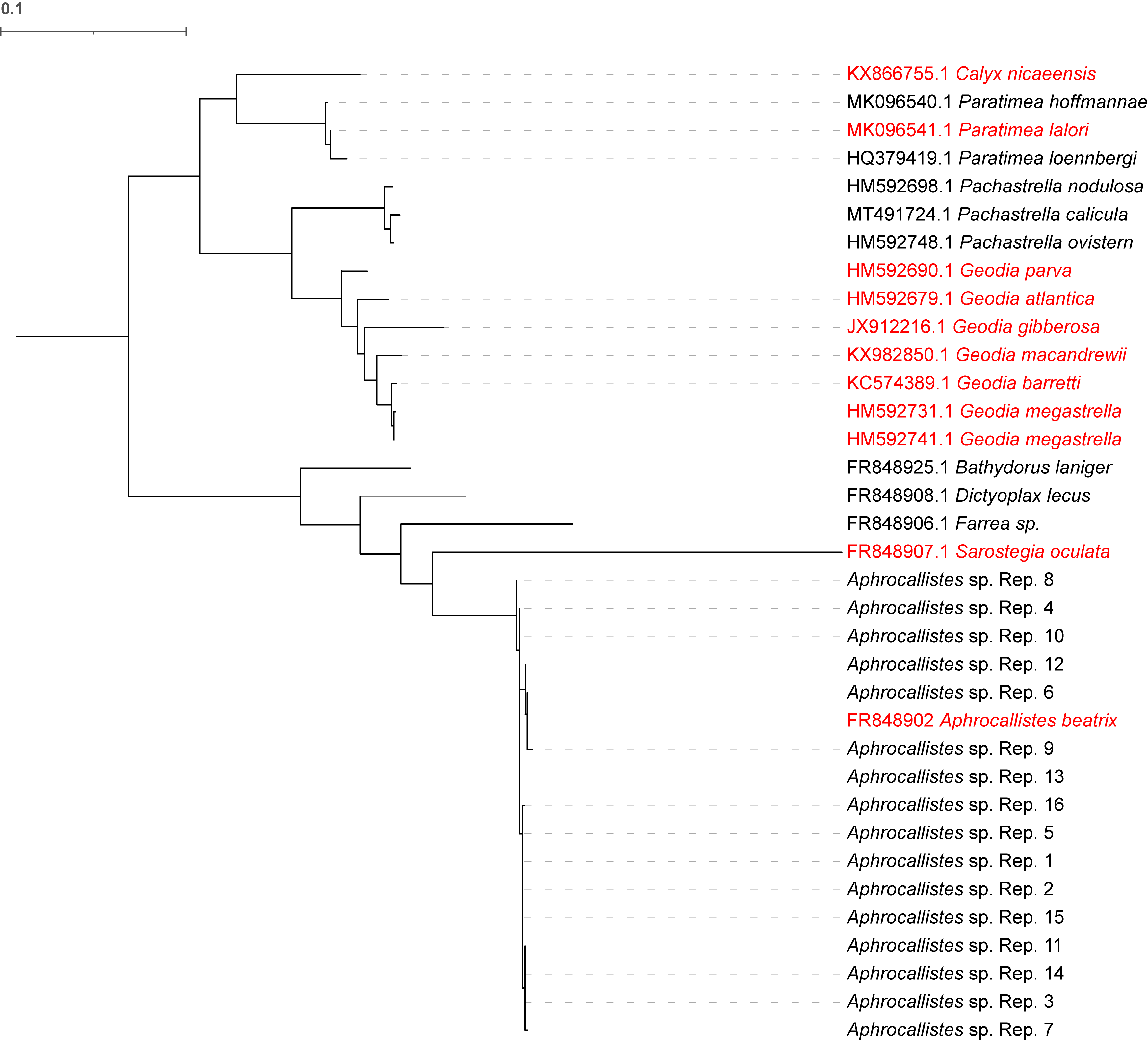


Supplementary Figure 2: Phylogenetic tree for *Paratimea* sp. samples based on part of the 28S rRNA gene using primer pairs C2 and D2. Reference sequences were obtained from NCBI. The tree is rooted with *Paramuricea clavata* (not shown here). Organisms in red represent voucher sequences deposited in NCBI. Scale bar represents the tree scale. Rep. stands for replicate.


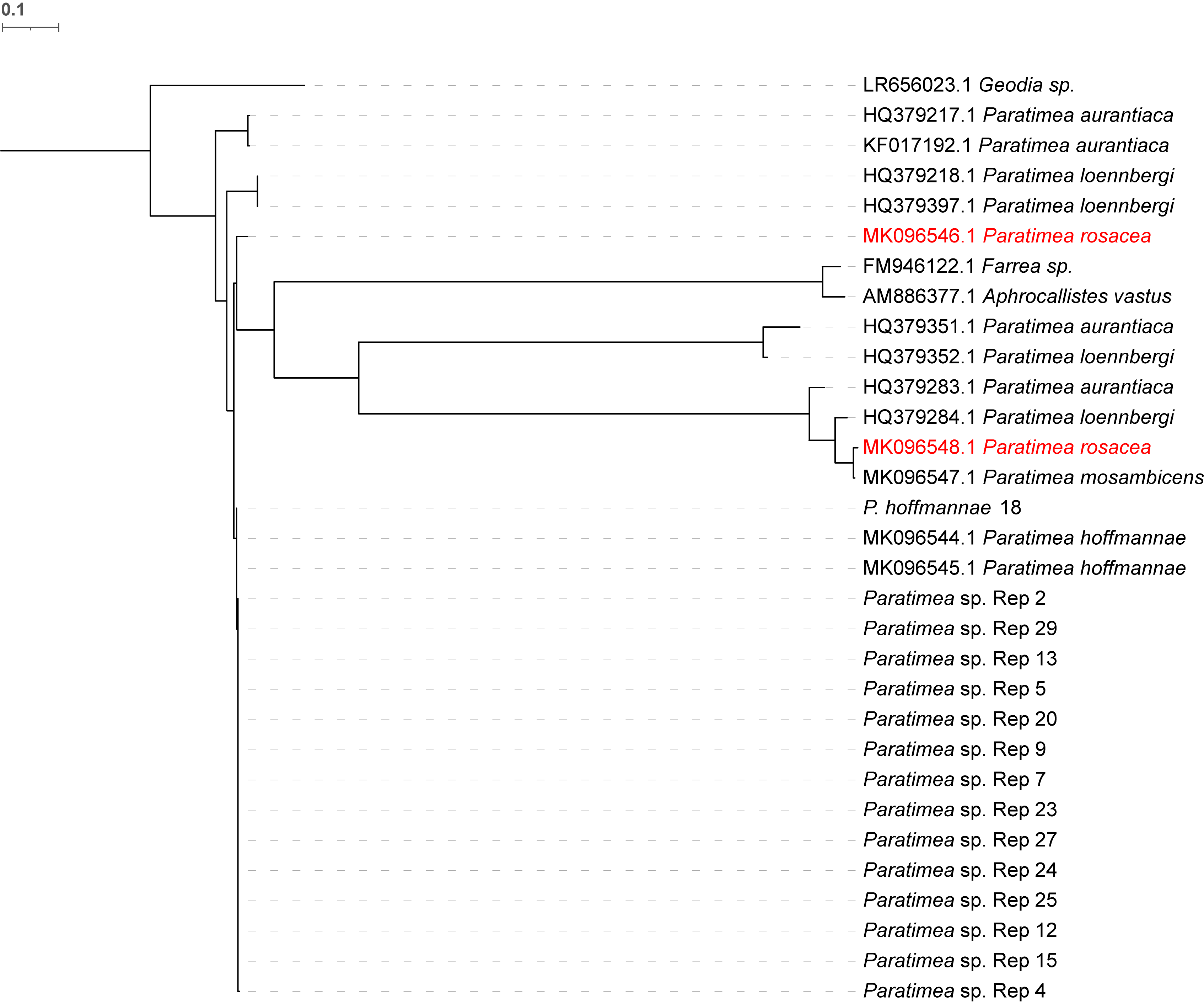


Supplementary Figure 3: Phylogenetic tree for *Pachastrella* sp. samples based on part of the COI gene using primer pairs dgLCO1490 and dgLCO2198. Reference sequences were obtained from NCBI. The tree is rooted with *Paramuricea clavata* (not shown here). Organisms in red represent voucher sequences deposited in NCBI. Scale bar represents the tree scale. Rep. stands for replicate.


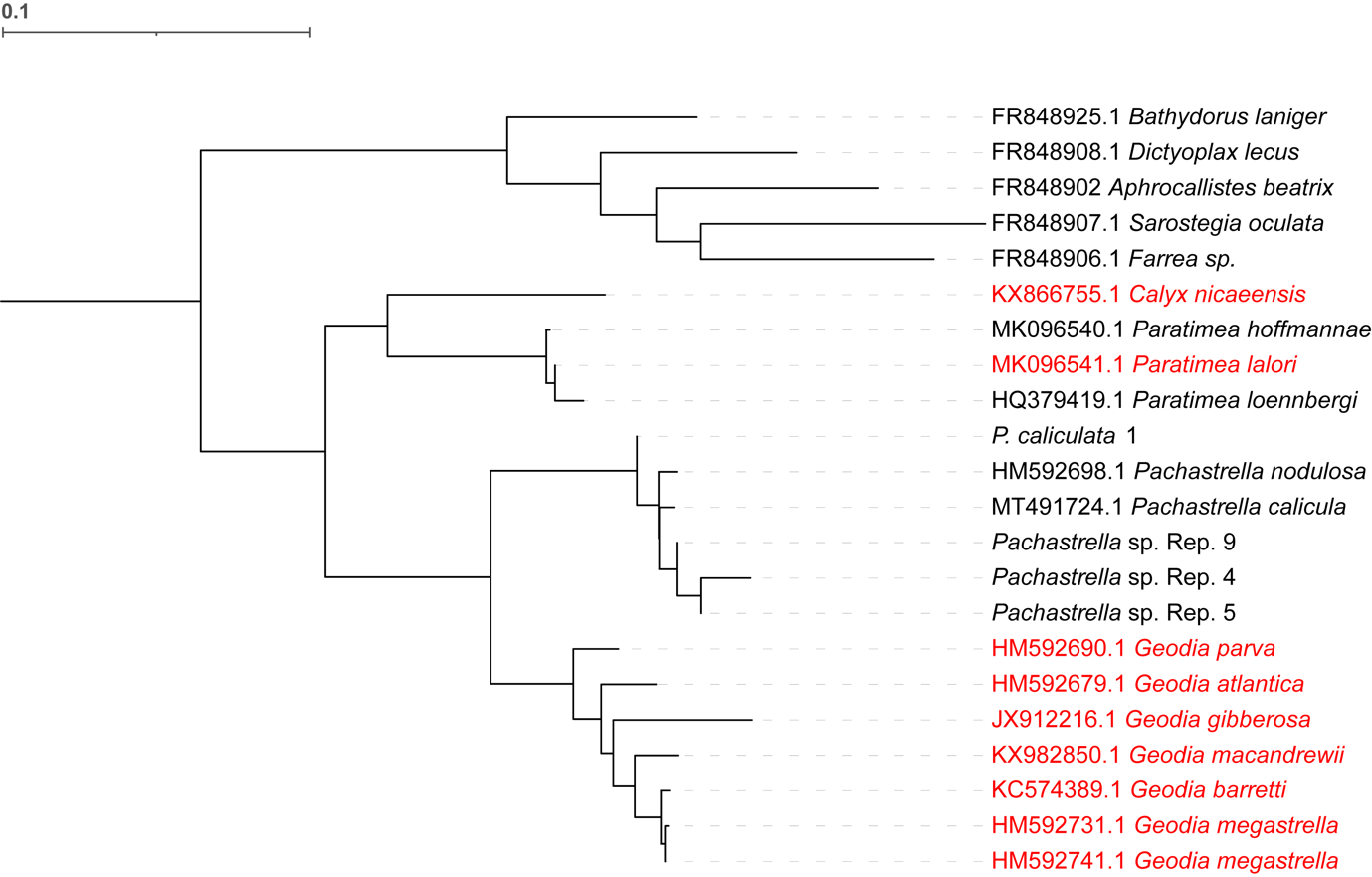


Supplementary Figure 4: Phylogenetic tree for *Geodia* sp. samples based on part of the COI gene using primer pairs dgLCO1490 and dgLCO2198. Reference sequences were obtained from NCBI. The tree is rooted with *Paramuricea clavata* (not shown here). Organisms in red represent voucher sequences deposited in NCBI. The remaining *Geodia* sp. samples were amplified using the primer pair CO1porF1 and CO1porR1 and were found to be identical to the sample shown here. Scale bar represents the tree scale. Rep. stands for replicate.


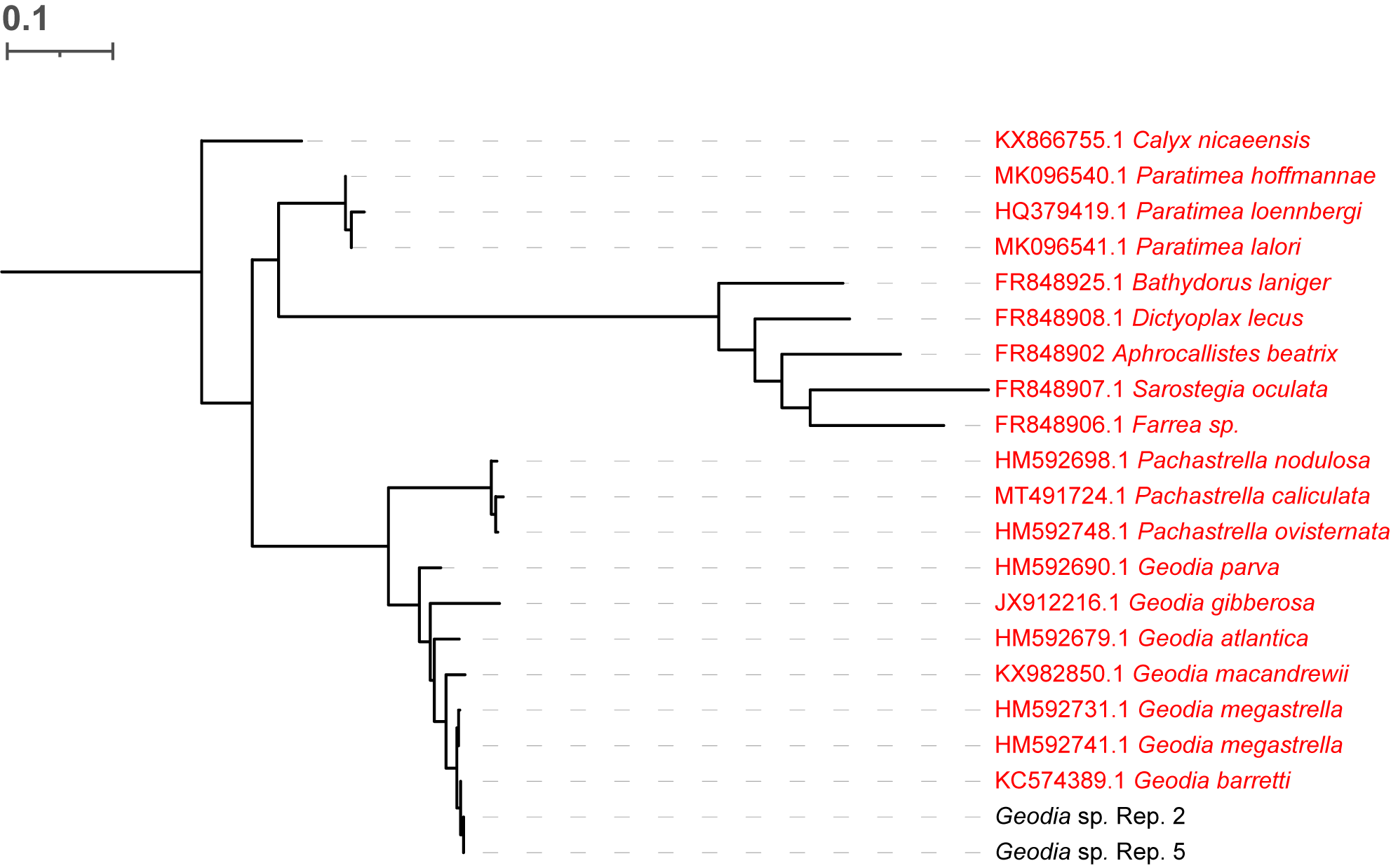


Supplementary Figure 5: Phylogenetic tree for *Farrea* sp*.* samples based on part of the COI gene using primer pairs dgLCO1490 and dgLCO2198. Reference sequences were obtained from NCBI. The tree is rooted with *Paramuricea clavata* (not shown here). Organisms in red represent voucher sequences deposited in NCBI. Scale bar represents the tree scale. Rep. stands for replicate.


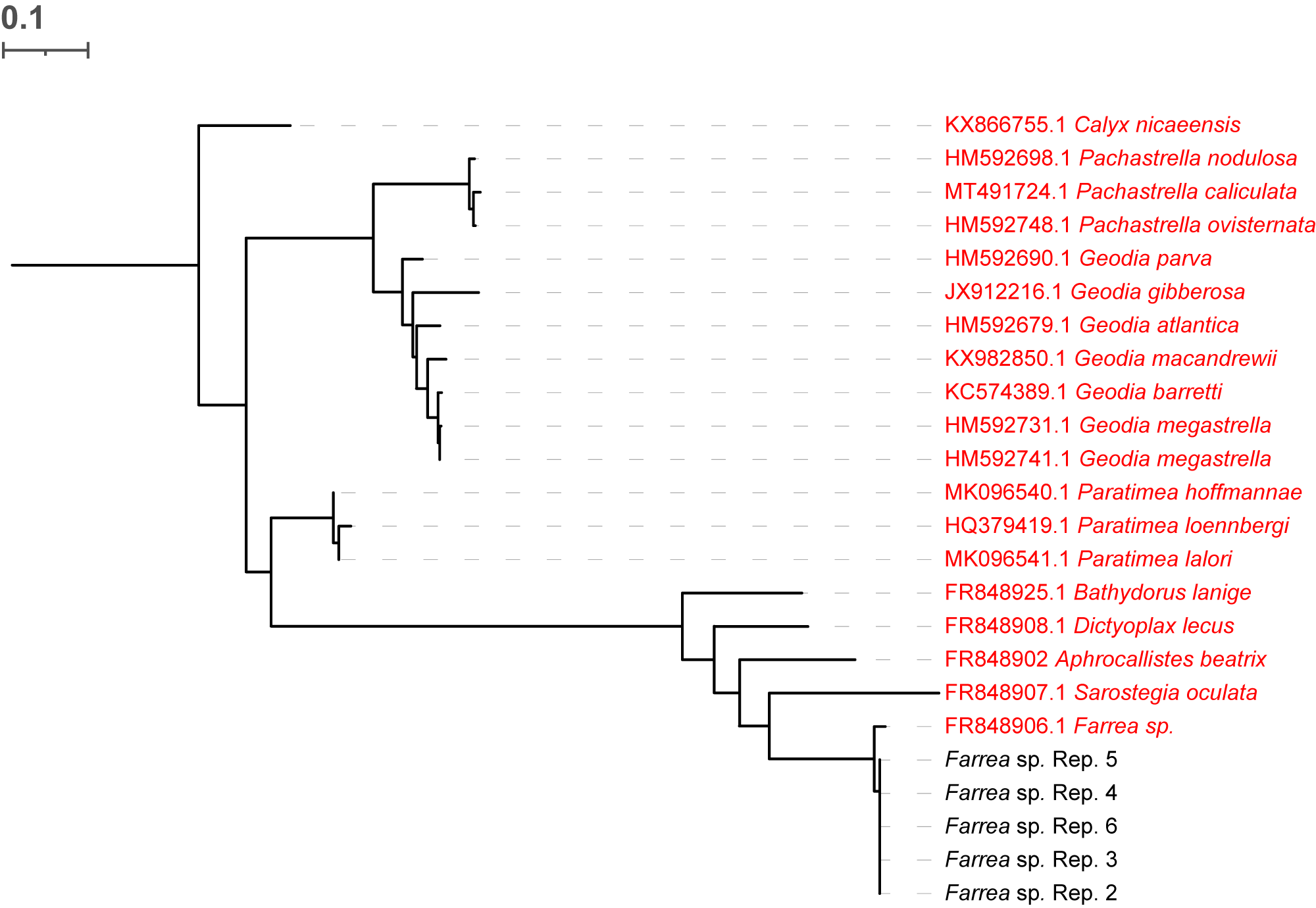


Supplementary Figure 6: Phylogenetic tree for *Calyx* sp. samples based on part of the COI gene using primer pairs dgLCO1490 and dgLCO2198. Reference sequences were obtained from NCBI. The tree is rooted with *Paramuricea clavata* (not shown here). Organisms in red represent voucher sequences deposited in NCBI. The remaining *Calyx* sp. samples were amplified using the 28S rRNA primer pairs C2 and D2 and were found to be identical to the sample shown here. Scale bar represents the tree scale. Rep. stands for replicate.


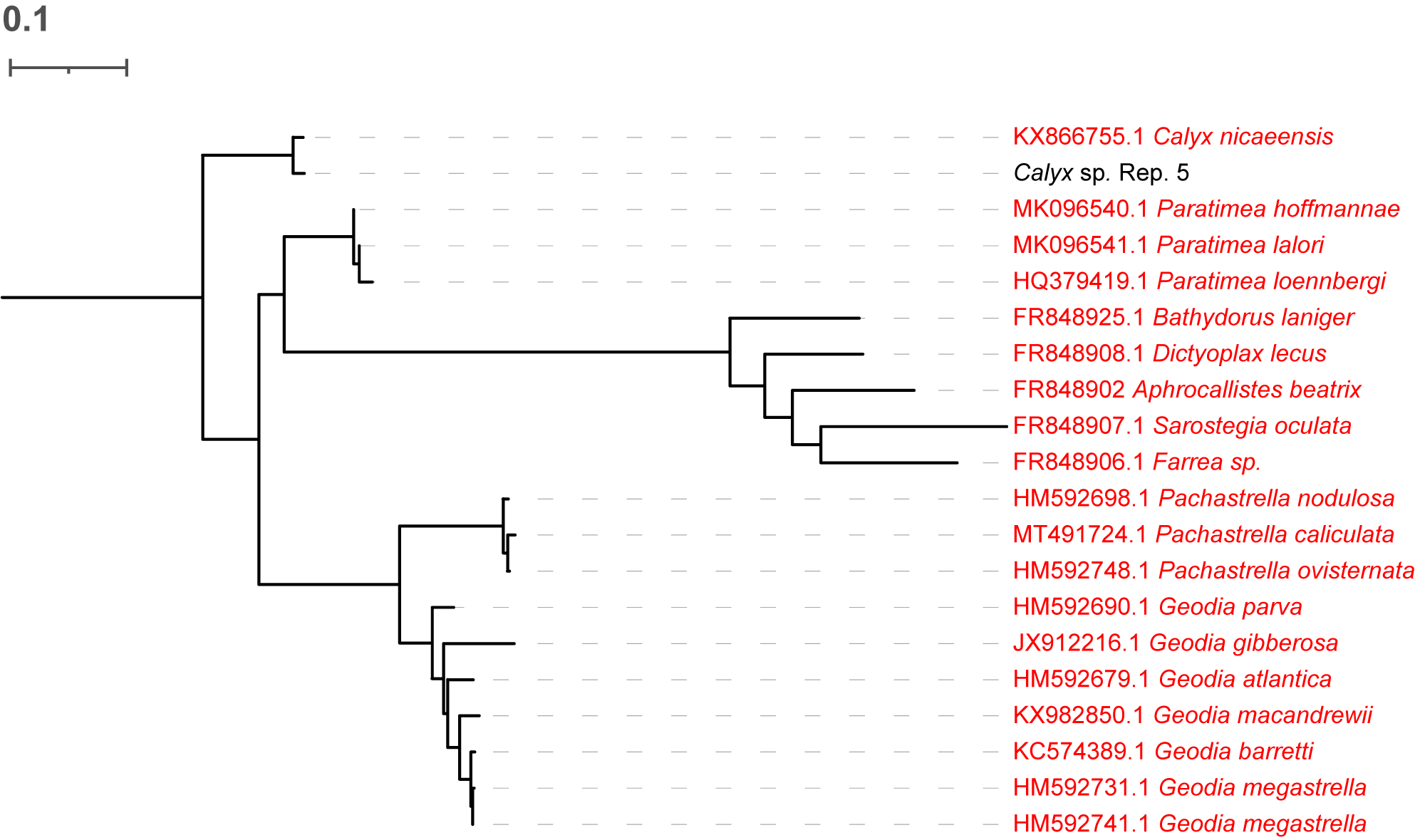


Supplementary Figure 7: Non-metric multidimensional scaling of the Bray-Curtis dissimilarity of microbial communities from different sponges, sediment and seawater.


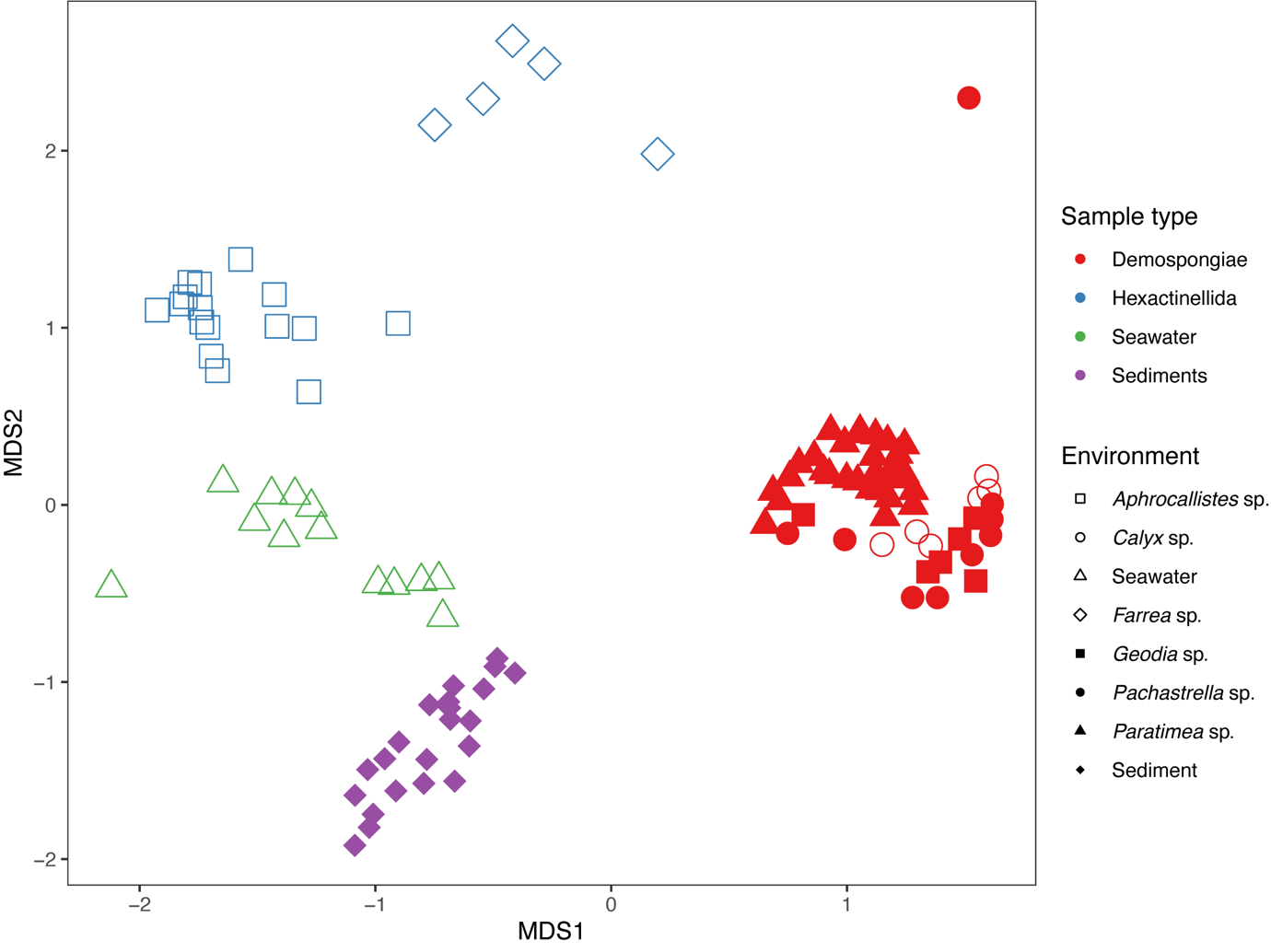

Supplement: Supplementary file 2 — Supplementary Figures [file 41396_2023_1439_MOESM2_ESM.docx]
